# Gabapentin for acute pain in sickle cell disease: A randomized double‐blinded placebo‐controlled phase II clinical trial

**DOI:** 10.1002/jha2.188

**Published:** 2021-05-04

**Authors:** Latika Puri, Kerri Nottage, Jane S. Hankins, Winfred C. Wang, Olivia McGregor, Jeffrey M. Gossett, Guolian Kang, Doralina L. Anghelescu

**Affiliations:** ^1^ Department of Hematology St. Jude Children's Research Hospital Memphis Tennessee USA; ^2^ Janssen Research and Development Raritan New Jersey USA; ^3^ Department of Biostatistics St. Jude Children's Research Hospital Memphis Tennessee USA; ^4^ Division of Anesthesia Department of Pediatric Medicine St. Jude Children's Research Hospital Memphis Tennessee USA; ^5^ Division of Pediatric Hematology/Oncology Department of Pediatrics Loma Linda University Children's Hospital Loma Linda California USA

**Keywords:** gabapentin, neuropathic pain, randomized controlled trial, sickle cell disease

## Abstract

Pain in sickle cell disease (SCD) can have a neuropathic component. This randomized phase II double‐blinded placebo‐controlled study evaluated the efficacy of gabapentin in reducing pain and opioid consumption (morphine‐equivalent dose [MED]) during acute vaso‐occlusive crisis (VOC). Of 90 patients aged 1–18 years with VOC pain, 45 were randomized to a single gabapentin dose (15 mg/kg) and 45 to placebo, in addition to standard treatment; 42 and 44 patients were evaluable in the gabapentin and placebo arms, respectively. A decrease in pain of ≥33% was reported in 68% of patients in the gabapentin arm and 60% of those in the placebo arm (one‐sided *p = *0.23). The median MED (mg/kg) in the gabapentin (0.12) and placebo arms (0.13) was similar (*p =* 0.9). However, in the subset of patients with the HbSS genotype (*n* = 45), the mean (SD) absolute pain score decrease by the time of discharge was significantly greater in the gabapentin arm (5.9 [3.5]) than in the placebo arm (3.6 [3.3]) (*p = *0.032). Pain scores in the overall study population were not significantly reduced when gabapentin was added to standard treatment; however, gabapentin benefited individuals with the more severe genotype, HbSS, during acute VOC. Larger, prospective studies are needed to confirm these findings.

## 1 INTRODUCTION

Acute vaso‐occlusive crisis (VOC) is the hallmark of sickle cell disease (SCD) and continues to be the most common cause of emergency room visits and hospitalizations in patients with SCD [1]. These pain crises increase in frequency, duration, and intensity with age [1]. Pain in SCD is typically considered nociceptive because of mechanisms of vaso‐occlusion, subsequent tissue ischemia, and inflammation [2, 3]. However, studies in mice with SCD implicate neuropathic pain mechanisms [4, 5, 6, 7, 8]. Additionally, patient‐reported outcome studies using neuropathic pain questionnaires suggest that 40% of adult patients with SCD endorse pain descriptors typically used by patients with known sources of neuropathic pain [3, 9, 10, 11]. Quantitative sensory testing studies in patients with SCD suggest both peripheral and central nervous system sensitization, whereas functional MRI findings suggest the presence of central nervous system abnormalities [9, 12, 13, 14, 15]. These data suggest a complex etiology of SCD pain, including ischemic, inflammatory, and neuropathic components. However, therapies targeting neuropathic pain in SCD have not been prospectively studied in the setting of acute pain crises.

The backbone of treatment of acute pain crisis has been intravenous opioids. Patients with SCD often require large opioid doses because of increased opioid metabolism and clearance [16], as well as tolerance from receiving long‐term opioid therapy, leading to pain that may become increasingly refractory to opioids [2].

Gabapentin has been used successfully to treat neuropathic pain in various clinical settings, including complex regional pain syndromes, diabetic neuropathy, and postherpetic neuralgia [17, 18, 19]. Additionally, it has proven opioid‐sparing effects in the acute pain postoperative pain setting in adults [20, 21] and children [22].

Gabapentinoids bind to presynaptic neurons at the alpha2‐delta subunit of voltage‐gated calcium channels and reduce calcium influx into presynaptic terminals and therefore reduce excessive release of excitatory neurotransmitters (e.g., glutamate, substance P, and noradrenaline); their effects on central sensitization and hyperalgesia are indirect rather than direct [23, 24]. Gabapentin and morphine may be synergistic because they act on independent targets within the peripheral and central nervous systems [25]. The mechanism for reducing central sensitization is reduction of hyperexcitability of secondary nociceptive neurons in the dorsal horn by suppressing the release of excitatory amino acids in the spinal cord in response to noxious stimuli [26]. In children, evidence for gabapentin use has been limited to case reports [27] and retrospective case series [28, 29, 30]; therefore, formally testing gabapentin in a prospective randomized study is warranted.

This randomized phase II double‐blinded placebo‐controlled study tested the hypothesis that gabapentin (in comparison to placebo) used in addition to standard treatment during an acute VOC in children with SCD would (1) reduce pain scores and (2) reduce opioid consumption.

## 2 PATIENTS AND METHODS

### 2.1 Enrollment and patient population characteristics

This randomized phase II double‐blinded placebo‐controlled trial was conducted at St. Jude Children's Research Hospital after approval by the Institutional Review Board (IRB). An Investigational New Drug approval (IND119125) was issued by the Food and Drug Administration. The study was registered at clinicaltrials.gov (NCT01954927). All authors had access to primary clinical trial data. The data analysis was performed by Jeffrey M Gossett and Guolian Kang. A full description of the study's methodology has been published [31]. Children and young adults between 1 and 18 years of age, with SCD (any genotype), who presented with VOC with a pain score >4, and required intravenous opioids but lacked other acute complications, such as acute chest syndrome or splenic sequestration, were eligible to participate. Patients were excluded for pain scores ≤4, renal dysfunction (GFR < 60 mL/min/1.73 m^2^), a history of seizures, current pregnancy or breastfeeding, or treatment with gabapentinoid or other anti‐epileptic drugs.

Patients were enrolled and randomized to receive either a single oral dose of gabapentin (15 mg/kg) or a similarly formulated dose of placebo in addition to standard treatment for VOC as per our institutional St. Jude Sickle Cell Disease Pain Management Guidelines. Standard treatment for acute VOC in patients at our institution includes intravenous hydration and analgesic management with nonsteroidal anti‐inflammatory drugs (ketorolac) and opioids (morphine or hydromorphone). In the acute care outpatient clinic, opioids are usually administered in a structured fashion (e.g., morphine 0.05–0.1 mg/kg, every 30 min as needed based on pain score, and the dose is titrated as clinically indicated, as presented in the supplemental material). For analysis, all opioid doses were converted to intravenous morphine equivalent doses (mg/kg) by opioid equianalgesic potency. The following ratios were used: fentanyl:morphine, 100:1; hydromorphone: morphine, 5:1 [32].

### 2.2 Clinical trial data sharing

Deidentified participant data and study protocol will be available beginning 9 months and ending 36 months following article publication. These data will be available to investigators whose proposed use of the data has been approved by an independent review committee identified for this purpose. Requests should be directed to the corresponding author; to gain access, data requestors will need to sign a data access agreement.

### 2.3 Primary outcome: pain score reduction

**FIGURE 1:**
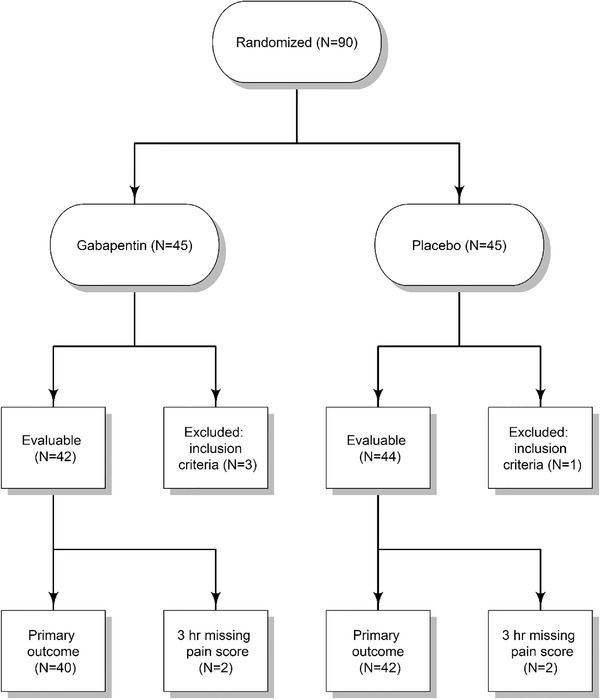
Consort flow diagram

The primary endpoint was defined as *a* ≥ 33% decline in pain score between baseline and assessment at 3 h poststudy drug administration. Pain scores were collected at four time points: *T*_0_, baseline (i.e., presentation to the acute care setting); *T*_1_, at the time of study drug administration; *T*_2_, 3 h poststudy drug administration; and *T*_3_, at the time of decision for disposition from the acute care setting either to discharge home or to admission to the hospital (Figure 1). Pain scores were documented in the electronic medical record. Pain was measured by using validated age‐appropriate pain scales: the FLACC (Faces, Legs, Arms, Cry, and Consolability) scale for children younger than 4 years [33]; the FACES scale for children aged from 4 to 7 years [34]; and the numeric rating scale for children older than 7 years [35, 36]. We analyzed the differences between the pain score at baseline (*T*_0_) and that at each of the other time points (*T*_1_, *T*_2_, *T*_3_). Pain score outcomes were examined for the overall study group.

### 2.4 Secondary outcome: opioid consumption

The opioid consumption in morphine‐equivalent dose (MED) during the duration of VOC from presentation (*T*_0_) to 3 h post‐study drug administration (*T*_2_) in the gabapentin group was compared with that in the placebo group.

### 2.5 Exploratory outcome: admission rates

Admission rates were examined in the two treatment arms. All outcomes were examined for the overall study group and the subgroup of patients with the HbSS genotype.

### 2.6 Statistical analysis

The study was designed to have no more than a 5% chance (*α* = 0.05) of falsely concluding that adding gabapentin to standard treatment increases the proportion of patients with a decrease in pain score when there is no difference between gabapentin and placebo groups and to have at least an 80% ( = 1 − *β*) power to correctly conclude that gabapentin increases the proportion of patients with a decrease in pain score from 45% to 65%. Block randomization with block sizes of four and six was used to randomize participants stratified by age and baseline pain score [31]. The intention‐to‐treat principle was followed, and all eligible subjects were analyzed in the primary analysis as randomized. Patient characteristics, pain assessments, and other outcomes were summarized and compared between study drug and placebo groups. Continuous variables were summarized as mean (standard deviation) or median (interquartile range). Categorical variables were summarized by the percentage of frequency. Continuous variables were compared by using either two‐group *t*‐tests or the nonparametric Wilcoxon/Mann‐Whitney tests, depending on the result of the Shapiro‐Wilks test of normality. Categorical variables were compared by using either Pearson's chi‐square or Fisher's exact test. Two‐sided tests were used for all comparisons other than the primary hypothesis. The primary outcome, a 33% decrease of pain score from presentation (*T*_0_) to 3 h after study drug administration (*T*_2_), was analyzed by using a right‐tailed (i.e., one‐sided) *z*‐test for proportions.

Statistical analyses were performed by using SAS software, version 9.4 (SAS Institute, Cary, NC) and RStudio (RStudio Team (2018); RStudio: Integrated Development for R. RStudio, Inc., Boston, MA) with R version 3.5.2 (R Core Team, Vienna, Austria). A *p‐*value < 0.05 was considered statistically significant.

## 3 RESULTS

### 3.1 Enrollment and patient population characteristics

The accrual goal was 166 patients, with interim analysis planned after half of the patients were enrolled. The results presented are from the interim analysis, with a total of 90 patients enrolled over 56 months (10/2013–05/2018). According to the study design, based on the interim analysis, the study was closed due to clinical futility based on lack of significant results of the primary pain score outcome.

**FIGURE 2:**
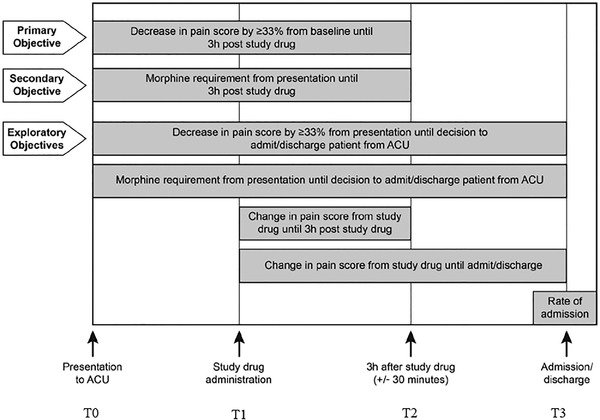
Study design

Participants’ demographics are summarized in Table 1. The mean (SD) age was 11.8 (4.9) years (range: 1.9–18.6 years). Gender distribution was similar in the two arms. HbSS was the predominant genotype in both groups. Forty‐five patients were randomized to each arm. Three patients in the gabapentin group were excluded from the analysis (one refused participation after consent and randomization; another refused to take the medicine due to unpalatability, and one patient's pain was determined to be caused by something other than VOC following consent and randomization). In the placebo group, one patient was excluded from analysis because the study drug arrived late, outside the time window allowed by the study design. Eighty‐six participants were evaluable: 42 in the gabapentin group and 44 in the placebo group (Figure 2). The power to detect the increase in the proportion of participants with ≥33% reduction in pain scores from 45% to 65% at a level of *p*‐value of 0.05 was 58%. This was based on a one‐sided two‐sample test for 90 patients (45 in each arm).

**TABLE 1:** Baseline demographics of study participants

Characteristic	All *N *= 86	Gabapentin arm *N* = 42	Placebo arm *N* = 44	*p*‐value
Age, mean (SD), year	11.8 (4.9)	11.8 (4.5)	11.8 (5.3)	0.77
Median (IQR)	12.4 (8.1, 15.8)	11.9 (8.2, 15.6)	14.3 (7.7, 16.3)	
Sex, *n* (%)				0.07
Male	51 (59.3%)	29 (69.0%)	22 (50.0%)	
Female	35 (40.7%)	13 (31.0%)	22 (50.0%)	
Sickle cell genotype, *n* (%)				0.019
HbSS	44 (51.2%)	18 (42.9%)	26 (59.1%)	
HbSC	25 (29.1%)	10 (23.8%)	15 (34.1%)	
HbS/β^0^ thalassemia	8 (9.3%)	6 (14.3%)	2 (4.5%)	
Other	9 (10.5%)	8 (19.0%)	1 (2.3%)	
Pain score on presentation				0.77
Mean (SD)	7.8 (1.8)	8.0 (1.6)	7.7 (1.9)	
Median (IQR)	8 (7, 10)	8 (7, 10)	8 (6.5, 10)	

**Abbreviation**: IQR, interquartile range.

### 3.2 Pain score reduction

Pain scores at presentation were similar for each group (*p *= 0.77), with a median (interquartile range [IQR]) of 8 (7–10) in the gabapentin arm and 8 (6.5–10) in the placebo arm. The primary outcome (i.e., ≥33% reduction in pain scores from *T*_0_ to *T*_2_) was achieved in 67.5% (27/40) of patients in the gabapentin arm and 59.5% (25/42) of patients in the placebo arm (one‐sided *p *= 0.23, Table 2). A favorable response (≥33% reduction in pain scores) from presentation (*T*_0_) to discharge from the acute care setting (*T*_3_) was noted in 75% (30 of 40) of patients in gabapentin arm and 61.4% (27 of 44) in the placebo arm (*p= *0.18) (Table 2). The absolute decrease in pain score from baseline (*T*_0_) to 3 h post‐treatment (*T*_2_) was not significantly different in the two arms (*p* = 0.25), and the median (IQR) decrease was 4.0 (1.5–7.0) in the gabapentin arm and 3.0 (1.0–6.0) in the placebo arm.

**TABLE 2:** Pain reduction from presentation to given time points for the entire cohort

Pain response	Gabapentin arm *N* = 42	Placebo arm *N* = 44	*p*‐value
≥33% reduction in pain scores from baseline to 3 h posttreatment	67.5% (27/40)	59.5% (25/42)	0.23^†^
≥33% reduction in pain scores from baseline to disposition from the acute care clinic	75.0% (30/40)	61.4% (27/44)	0.18
Decrease in pain score from baseline to 3 h posttreatment			0.25
*N*	40	42	
Mean (SD)	4.2 (3.3)	3.3 (3.0)	
Median (IQR)	4.0 (1.5, 7.0)	3.0 (1.0, 6.0)	
Decrease in pain score from baseline to disposition from acute care setting			0.24
*N*	40	44	
Mean (SD)	4.5 (3.4)	3.6 (3.3)	
Median (IQR)	4.0 (2.0, 7.0)	3.0 (1.0, 6.5)	

^†^ One‐sided test

**Abbreviation**: IQR, interquartile range.

We performed a sub‐analysis of participants with the most severe sickle genotype, HbSS. Although the proportion of patients with HbSS in whom the primary endpoint was achieved was higher in the gabapentin arm than in the placebo arm (76.5% [13 of 17] in HbSS and 61.5% [16 of 26] in the placebo arm), the difference was not statistically significant (*p = *0.31) (Table 3). The absolute decrease in pain score of HbSS participants from presentation (*T*_0_) to 3 h poststudy drug administration (*T*_2_) was not significantly different between treatment arms but may suggest a trend, with mean (SD) pain score decrease of 5.5 (3.4) and 3.6 (3.1) in gabapentin and placebo arms, respectively (*p = *0.061). Importantly, the mean (SD) absolute decrease in pain score from presentation (*T*_0_) to discharge from the acute care setting (*T*_3_) was significantly higher in the gabapentin group (5.9 [3.5]) than in the placebo group (3.6 [3.3]) (*p = *0.032).

**TABLE 3:** Pain reduction in HbSS subgroup

Pain response	Gabapentin arm *N* = 17	Placebo arm *N* = 26	*p*‐value
≥33% reduction in pain scores from baseline to 3 h posttreatment	76.5% (13/17)	61.5% (16/26)	0.31
≥33% reduction in pain scores from baseline to disposition from the acute care clinic	88.2% (15/17)	61.5% (16/26)	0.085
Decrease in pain score from baseline to 3 h posttreatment			0.061
*N*	17	26	
Mean (SD)	5.5 (3.4)	3.6 (3.1)	
Median (IQR)	6.0 (4,0, 8.0)	3.0 (1.0, 5.0)	
Decrease in pain score from baseline to disposition from acute care setting,			**0.032**
*N*	17	26	
Mean (SD)	5.9 (3.5)	3.6 (3.3)	
Median (IQR)	6.0 (4.0, 8.0)	3.0 (1.0, 7.0)	

**Abbreviation**: IQR, interquartile range.

Bold values show statistically significant *p*‐value.

### 3.3 Opioid consumption

Opioid consumption, as measured by MED (mg/kg), from presentation (*T*_0_) to 3 h poststudy drug administration (*T*_2_) was similar between the two treatment groups (*p *= 0.90). The median (IQR) MED was 0.12 mg/kg (0.09–0.22 mg/kg) in the gabapentin arm and 0.13 mg/kg (0.09–0.22 mg/kg) in the placebo arm. Likewise, MED for the HbSS subgroup did not differ significantly between treatment arms, with a median (IQR) MED of 0.11 mg/kg (0.09–0.23 mg/kg) in the gabapentin arm and 0.12 mg/kg (0.08–0.23 mg/kg) in the placebo arm *(p = *0.93).

### 3.4 Admission rates

As an exploratory objective, we evaluated admission rates in the study arms. A similar number of patients in each group were hospitalized for further pain management: 24% (10 of 42) in the gabapentin arm and 27.3% (12 of 44) in the placebo arm (*p = *0.71). In the HbSS genotype subgroup, 34.6% of patients (9 of 26) in the placebo arm and 11.1% of patients (2 of 18) in the gabapentin arm were admitted (*p = *0.16) (Table S2).

A combined analysis of participants with HbSS and HbS/β^0^ thalassemia did not yield any significant differences in the primary or secondary outcomes between the gabapentin and placebo groups.

### 3.5 Adverse effects

Adverse effects noted in the gabapentin arm included vomiting (*n* = 2), fatigue (*n* = 1), pneumonitis (*n* = 1), itching (*n* = 1), dizziness (*n* = 1), and headache (*n* = 1). Adverse effect in the placebo arm included vomiting (*n* = 1) and pneumonitis (*n* = 1). All were grade 1 and no serious adverse effects were noted.

## 4 DISCUSSION

Although neuropathic pain is a known component of VOC, drugs targeting neuropathic pain have not been systematically studied in patients with SCD. Preliminary studies and case reports suggest some efficacy of treatments in reducing the frequency of VOC [37, 38]. Our study is the first prospective randomized trial investigating the efficacy of gabapentin during acute VOC pain in SCD. Through a controlled study, we evaluated the utility of adding gabapentin to the standard management of VOC in SCD. A single high dose of gabapentin (15 mg/kg) was chosen for this trial because studies suggest that single high‐dose gabapentin can lead to a significant decrease in pain score and opioid consumption [39, 40, 41, 42, 43], whereas low‐dose gabapentin has limited efficacy [44].

Although the primary endpoint did not reach statistical significance for the entire study cohort, adding gabapentin to standard management of acute VOC for the subgroup of patients with the HbSS genotype resulted in a significantly greater reduction in pain scores from presentation to discharge from acute care setting when compared to the addition of placebo. We did not observe a significant difference between the two study groups for the secondary outcome of opioid consumption. This may be partially related to the fact that the opioid doses were administered using structured dose and time interval guidelines (morphine 0.05–0.1 mg/kg, every 30 min as needed), so access to opioids was standardized for all patients and did not allow the flexibility of self‐administration offered by a patient‐controlled analgesia design.

Gabapentin use did not influence the rate of hospitalization for the overall study group, as admission rates were similar for both study arms. Nevertheless, among those with the HbSS genotype, the admission rate was higher in the placebo arm than in the gabapentin arm, without reaching statistical significance.

Based on the interim analysis results, and in accordance with the study design, the study team decided to stop enrollment due to clinical futility (i.e., unlikely to meet the primary endpoint even with full accrual). While the study closed early for futility and had only modest efficacy, it is an important study advancing the use of alternative analgesics for acute SCD‐related pain.

Some limitations of our study warrant discussion. The study population was small, and our target enrollment was not met due to several challenges common to research in an acute pain setting in patients with SCD. Study enrollment was limited to regular clinic hours and research personnel generally were not available after hours. Participants were allowed enrollment in the study only once. Thus, the overall eligible study population was limited and exhausted after a few years of ongoing study because, in general, the same subgroup of SCD patients tend to have repeated VOC‐related visits. Furthermore, only a short time window was allotted for the administration of study drug (i.e., within 1 h of initial opioid administration), which was logistically challenging. Another challenge was obtaining consent from children and young adults who presented in distress, which made eliciting assent from children and consent among those at the age of majority problematic. Occasionally, patients had received opioids and were sleepy before being approached for enrollment. To overcome this, we worked closely with the IRB to include provisions protecting patients under these circumstances. Approval was obtained to allow candidates 18 years or older to consent via surrogate decision‐makers whenever they believed their pain experience or the effects of the pain medications were hindering their ability to give consent independently. An additional challenge was posed by enrollment limited to a single institution, which reduced the patient pool from which the subjects were recruited. Children with SCD have elevated GFR [45]. Thus, they may have lower serum gabapentin serum levels. Therapeutic dose ranges of gabapentin in SCD patients are unknown and sub‐therapeutic dosing in our study could have been a possible reason for lack of efficacy.

Our study design was based on the assumption that patients studied had a neuropathic pain component. A better study design may be focused on studying the efficacy of gabapentin in patients with and without neuropathic pain, as determined by clinical neuropathic pain assessment tools such as PainDetect [46].

Overall, our study was a rigorously conducted randomized double‐blinded placebo‐controlled trial with an objective pain reduction end point. All VOC episodes were treated in a standardized fashion followed with high fidelity.

Gabapentin could be a useful adjunct to standard management for patients with the HbSS genotype, but future studies in a larger cohort of patients with the HbSS genotype and known neuropathic pain might better evaluate gabapentin's utility. The authors assumed that patients studied had a neuropathic pain component. Future studies should focus on patients with known neuropathic SCD‐related pain and compare the efficacy of gabapentin in patients with and without neuropathic pain.

## AUTHOR CONTRIBUTIONS

Latika Puri, Doralina L. Anghelescu, Kerri Nottage, Jane S. Hankins, Winfred C. Wang, Guolian Kang, and Jeffrey M. Gossett designed the study, analyzed the data, and contributed to the manuscript. Olivia McGregor assisted with study coordination.

## CONFLICT OF INTEREST

D.L.A., L.P., W.C.W., G.K., J.M.G., and O.M. have no conflict of interest. J.S.H. receives research support from Global Blood Therapeutics and consultant fees from Global Blood Therapeutics and MJ Lifesciences. K.N. is a Janssen R&D employee and a JNJ stockholder.

## Supporting information


**Supplemental Table 2**. Admission rates at the end of acute care visit for the entire cohort and HbSS subgroups.
