# Supplementary material for: Gabapentin for acute pain in sickle cell disease: A randomized double‐blinded placebo‐controlled phase II clinical trial
Source: EJHaem. 2021 May 4;2(3):327–34. doi: 10.1002/jha2.188 (PMC9175868; doi:10.1002/jha2.188)
Supplement: Supplementary file 1 — Supplemental Table 2. Admission rates at the end of acute care visit for the entire cohort and HbSS subgroups. [file JHA2-2-327-s001.docx]

**Supplemental Table 2.**

Admission rates at the end of acute care visit for entire cohort and HbSS subgroups.

| All patients | | | |
| --- | --- | --- | --- |
|  | Gabapentin  N = 42 | Placebo  N= 44 | *P-*value |
| Number of patients hospitalized | 10 (23.8%) | 12 (27.3%) | 0.71 |
| **HbSS sub group** | | | |
|  | Gabapentin  N = 18 | Placebo  N= 26 |  |
| Number of patients hospitalized | 2 (11.1%) | 9 (34.6%) | 0.16 |
